# Predicting commercially available antiviral drugs that may act on the novel coronavirus (SARS-CoV-2) through a drug-target interaction deep learning model

**DOI:** 10.1016/j.csbj.2020.03.025

**Published:** 2020-03-30

**Authors:** Bo Ram Beck, Bonggun Shin, Yoonjung Choi, Sungsoo Park, Keunsoo Kang

**Affiliations:** a Deargen, Inc., Daejeon, Republic of Korea; b Department of Computer Science, Emory University, Atlanta, GA, United States; c Department of Microbiology, College of Science & Technology, Dankook University, Cheonan, Republic of Korea

**Keywords:** COVID-19, SARS-CoV-2, Coronavirus, MT-DTI, Deep learning, Drug repurposing, Atazanavir

## Abstract

The infection of a novel coronavirus found in Wuhan of China (SARS-CoV-2) is rapidly spreading, and the incidence rate is increasing worldwide. Due to the lack of effective treatment options for SARS-CoV-2, various strategies are being tested in China, including drug repurposing. In this study, we used our pre-trained deep learning-based drug-target interaction model called Molecule Transformer-Drug Target Interaction (MT-DTI) to identify commercially available drugs that could act on viral proteins of SARS-CoV-2. The result showed that atazanavir, an antiretroviral medication used to treat and prevent the human immunodeficiency virus (HIV), is the best chemical compound, showing an inhibitory potency with *K_d_* of 94.94 nM against the SARS-CoV-2 3C-like proteinase, followed by remdesivir (113.13 nM), efavirenz (199.17 nM), ritonavir (204.05 nM), and dolutegravir (336.91 nM). Interestingly, lopinavir, ritonavir, and darunavir are all designed to target viral proteinases. However, in our prediction, they may also bind to the replication complex components of SARS-CoV-2 with an inhibitory potency with *K_d_* < 1000 nM. In addition, we also found that several antiviral agents, such as Kaletra (lopinavir/ritonavir), could be used for the treatment of SARS-CoV-2. Overall, we suggest that the list of antiviral drugs identified by the MT-DTI model should be considered, when establishing effective treatment strategies for SARS-CoV-2.

## Highlights

• The MT-DTI deep learning model was used to identify potent drugs for SARS-CoV-2.
• Atazanavir, remdesivir, and Kaletra were predicted to inhibit SARS-CoV-2.
• Rapamycin and tiotropium bromide may also be effective for SARS-CoV-2.

## 1 Introduction

Coronaviruses (CoVs), belonging to the family *Coronaviridae*, are positive-sense enveloped RNA viruses and cause infections in birds, mammals, and humans [1], [2], [3]. The family includes four genera, such as *Alphacoronavirus*, *Betacoronavirus*, *Deltacoronavirus*, and *Gammacoronavirus* [4]. Two infamous infectious coronaviruses in the genus *Betacoronavirus* are severe acute respiratory syndrome coronavirus (SARS-CoV) [5] and Middle East respiratory syndrome coronavirus (MERS-CoV) [6], which have infected more than 10,000 people around the world in the past two decades. Unfortunately, the incidence was accompanied by high mortality rates (9.6% for SARS-CoV and 34.4% for MERS-CoV), indicating that there is an urgent need for effective treatment at the beginning of the outbreak to prevent the spread [7], [8]. However, this cannot be achieved with current drug development or an application system, taking several years for newly developed drugs to come to the market. Unexpectedly, the world is facing the same situation as the previous outbreak due to a recent epidemic of atypical pneumonia (designated as coronavirus disease 2019; COVID-19) caused by a novel coronavirus (severe acute respiratory syndrome coronavirus 2; SARS-CoV-2) in Wuhan, China [5], [9].

SARS-CoV-2, which belongs to *Betacoronavirus*, contains a positive-sense single-stranded RNA [(+)ssRNA] genome (29,903 bp) and contains genes encoding 3C-like proteinase, RNA-dependent RNA polymerase (RdRp), 2′-O-ribose methyltransferase, spike protein, envelope protein, nucleocapsid phosphoprotein, and several unknown proteins, according to the genome sequencing data of SARS-CoV-2 (https://www.ncbi.nlm.nih.gov/genbank/sars-cov-2-seqs/). Typical clinical symptoms of COVID-19 are fever, dry cough, and fatigue within 3–7 days of latency on average after infection. This is relatively slower than severe acute respiratory syndrome (SARS), which was caused by SARS-CoV [10]. During the life cycle of coronaviruses, the virus replicates via the following processes after entering the host cell: 1) translation of genomic RNA (gRNA), 2) proteolysis of the translated polyprotein with viral 3C-like proteinase, 3) replication of gRNA with the viral replication complex that consists of RNA-dependent RNA polymerase (RdRp), helicase, 3′-to-5′ exonuclease, endoRNAse, and 2′-O-ribose methyltransferase, and 4) assembly of viral components [11]. These replication-associated proteins are the primary targets of post-entry treatment drugs to suppress viral replication. Although much intensive effort is being made worldwide to develop drugs or vaccines for SARS-CoV-2, patients currently suffering from COVID-19 cannot expect benefits from them due to the slow development process of novel drugs or vaccines.

Thus, a rapid drug application strategy that can be immediately applied to the patient is necessary. Currently, the only way to address this matter is to repurpose commercially available drugs for the pathogen in so-called “drug-repurposing”. However, in theory, artificial intelligence (AI)-based architectures must be taken into account in order to accurately predict drug-target interactions (DTIs). This is because of the enormous amount of complex information (e.g. hydrophobic interactions, ionic interactions, hydrogen bonding, and/or van der Waals forces) between molecules. To this end, we previously developed a deep learning-based drug-target interaction prediction model, called Molecule Transformer-Drug Target Interaction (MT-DTI) [12].

In this study, we applied our pre-trained MT-DTI model to identify commercially available antiviral drugs that could potentially disrupt SARS-CoV-2′s viral components, such as proteinase, RNA-dependent RNA polymerase, and/or helicase. Since the model utilizes simplified molecular-input line-entry system (SMILES) strings and amino acid (AA) sequences, which are 1D string inputs, it is possible to quickly apply target proteins that do not have experimentally confirmed 3D crystal structures, such as viral proteins of SARS-CoV-2. We share a list of top commercially available antiviral drugs that could potentially hinder the multiplication cycle of SARS-CoV-2 with the hope that effective drugs can be developed based on these AI-proposed drug candidates and act against SARS-CoV-2.

## 2 Matereials & methods

### 2.1 Amino acid sequences used in this study

Amino acid sequences of 3C-like proteinase (accession YP_009725301.1), RNA-dependent RNA polymerase (accession YP_009725307.1), helicase (accession YP_009725308.1), 3′-to-5′ exonuclease (accession YP_009725309.1), endoRNAse (accession YP_009725310.1), and 2′-O-ribose methyltransferase (accession YP_009725311.1) of the SARS-CoV-2 replication complex were extracted from the SARS-CoV-2 whole genome sequence (accession NC_045512.2), from the National Center for Biotechnology Information (NCBI) database.

### 2.2 Prediction of drug-target interactions using binding affinity scores

Molecule transformer-drug target interaction (MT-DTI) was used to predict binding affinity values between commercially available antiviral drugs and target proteins. MT-DTI is based on the self-attention mechanism that showed remarkable success in natural language process (NLP) literature. MT-DTI is inspired by the idea that for a chemist, understanding a molecule sequence is analogous to understanding a language. To apply the NLP model to drug-target interaction (DTI) tasks, MT-DTI is pre-trained with ‘chemical language’ (represented as SMILES) of approximately 1,000,000,000 compounds. Similar to the NLP model, which successfully extracts complex patterns from word sequences, MT-DTI successfully finds useful information in DTI tasks. Therefore, it shows the best performance and most robust results in diverse DTI datasets according to a previous study [12].

To train the model, the Drug Target Common (DTC) database [13] and BindingDB [14] database were manually curated and combined. Three types of efficacy value, *K_i_*, *K_d_*, and IC_50_ were integrated by a consistence-score-based averaging algorithm [15] to make the Pearson correlation score over 0.9 in terms of *K_i_*, *K_d_*, and IC_50_. Since the BindingDB database includes a wide variety of species and target proteins, the MT-DTI model has the potential power to predict interactions between antiviral drugs and SARS-CoV-2 proteins.

After the MT-DTI prediction, the raw prediction results were screened for antiviral drugs that are FDA approved, target viral proteins, and have a *K_d_* value < 1000 nM. SMILES containing salt forms were excluded from the final results as the prediction is focused to pairs of a single molecule and the target protein. In addition, remdesivir was also incoprated in the analysis as its therapeutic potential to COVID-19 is recently suggested by Wang et al. [16] and Gliead Sciences announcements (https://www.gilead.com/purpose/advancing-global-health/covid-19).

### 2.3 Prediction of drug-target interactions using AutoDock Vina

AutoDock Vina (version 1.1.2), which is a molecular docking and virtual screening application [17], was used to predict binding affinities (kcal/mol) between 3C-like proteinase of SARS-CoV-2 and 3,410 FDA-approved drugs. SMILES of 3,410 FDA-approved drugs were converted to the PDBQT format using Open Babel (version 2.3.2) [18] with the following options: --gen3d and -p 7.4. The hydrogens were added to the 3C-like proteinase model using MGLTools (version 1.5.6) [19]. Then, binding affinities between the protein and FDA-approved drugs were calculated using AutoDock Vina. The exhaustiveness parameter was set to 10.

## 3 Results

**Fig. 1:**
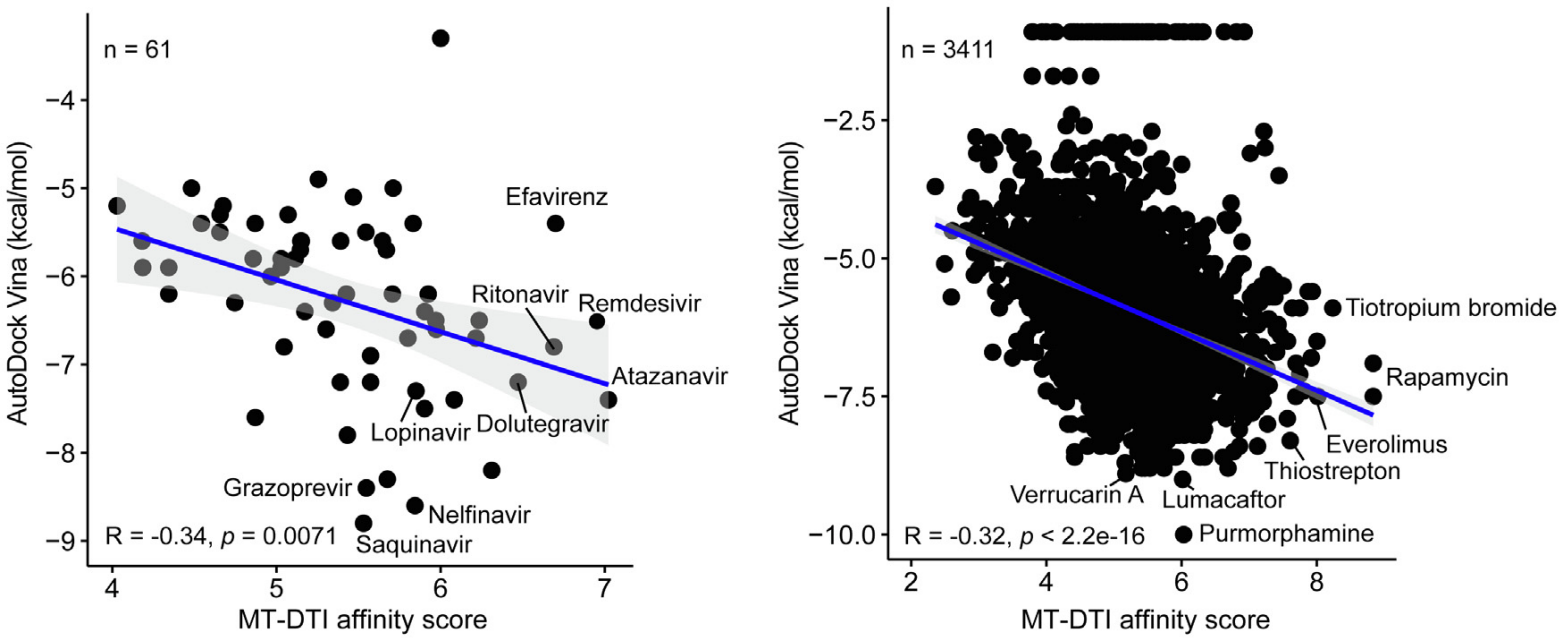
Comparison of MT-DTI and AutoDock Vina results. 60 known FDA-approved antiviral drugs (left) and 3410 FDA-approved drugs (right) were evaluated by means of the MT-DTI deep learning-based affinity score (higher is better), and AutoDock Vina docking score (lower is better). Remdesivir, which is not an FDA-approved drug, but regarded as a promising antiviral drug for SARS-CoV-2, was included in this analysis.

To identify potent FDA-approved drugs that may inhibit the functions of SARS-CoV-2′s core proteins, we used the MT-DTI deep learning-based model, which can accurately predict binding affinities based on chemical sequences (SMILES) and amino acid sequences (FASTA) of a target protein, without their structural information [12]. This deep learning-based approach is particularly useful, since it does not require protein structural information, which can be a bottleneck for identifying drugs targeted for uncharacterized proteins with traditional three-dimensional (3D) structure-based docking approaches [20]. Neverthless, MT-DTI showed the best performance [12] when compared to a deep learning-based (DeepDTA) approach [21] and two traditional machine learning-based algorithms SimBoost [22], and KronRLS [23], with the KIBA [24] and DAVIS [25] data sets. Taking advantage of this sequence-based drug-target affinity prediction approach, binding affinities of 3,410 FDA-approved drugs against 3C-like proteinase, RdRp, helicase, 3′-to-5′ exonuclease, endoRNAse, and 2′-O-ribose methyltransferase of SARS-CoV-2 were predicted. To confirm the performance of MT-DTI at least *in silico*, we compared the binding affinities of 3,410 FDA-approved drugs predicted by MT-DTI to those estimated by AutoDock Vina (a widely used 3D structure-based docking algorithm). It was possible since the 3D structure of the 3C-like proteinase protein was recently unveiled by the X-ray crystallography (PDBID 6LU7) [26]. Significant negative correlations, meaning that the results of both algorithms showed moderate similarities (higher is better for MT-DTI, whereas lower is better for AutoDock Vina) were observed in both the antiviral drug dataset (R = −0.34, and *p*-value = 0.0071) and the FDA-approved drug dataset (R = −0.32, and *p*-value < 2.2e−16) (Fig. 1). While it is not possible to determine which algorithm is more reliable without various experimental evaluations, a previous study showed that the MT-DTI model is one of the best deep learning-based models that can predict the binding affinity between a given protein and compound [12]. Therefore, we further applied the MT-DTI model to repurpose those FDA-approved drugs that have the potential to inhibit key proteins of SARS-CoV-2.

The SARS-CoV-2 3C-like proteinase was predicted to bind with atazanavir (*K_d_* 94.94 nM), followed by remdesivir, efavirenz, ritonavir, and other antiviral drugs that have a predicted affinity of *K_d_* > 100 nM potency (Table 1). No other protease inhibitor antiviral drug was found in the *K_d_* < 1000 nM range. Although there is no real-world evidence about whether these drugs will act as predicted against COVID-19 yet, some case studies have been identified. For example, a docking study of lopinavir along with other HIV proteinase inhibitors of the CoV proteinase (PDBID 1UK3) suggests atazanavir and ritonavir, which are listed in the present prediction results, may inhibit the CoV proteinase in line with the inhibitory potency of lopinavir [27]. According to the prediction, viral proteinase-targeting drugs were predicted to act more favorably on the viral replication process than viral proteinase through the DTI model (Table 2, Table 3, Table 4, Table 5, Table 6). The results include antiviral drugs other than proteinase inhibitors, such as guanosine analogues (e.g., acyclovir, ganciclovir, and penciclovir), reverse transcriptase inhibitors, and integrase inhibitors.

**Table 1:** Drug-target interaction (DTI) prediction results of FDA approved antviral drugs available on markets against a novel coronavirus (SARS-CoV-2, NCBI reference sequence NC_045512.2) 3C-like proteinase (accession YP_009725301.1). * indicates isomeric form SMILES.

Small molecules	SMILES used in the DTI prediction	*K_d_* in nM	MT-DTI Rank out of 3411
Atazanavir	COC(=O)NC(C(=O)NC(Cc1ccccc1)C(O)CN(Cc1ccc(—c2ccccn2)cc1)NC(=O)C(NC(=O)OC)C(C)(C)C)C(C)(C)C	94.94	66
Remdesivir*	CCC(CC)COC(=O)[C@H](C)N[P@](=O)(OC[C@@H]1[C@H]([C@H]([C@](O1)(C#N)C2 = CC = C3N2N = CN = C3N)O)O)OC4 = CC = CC = C4	113.13	70
Efavirenz*	O = C1Nc2ccc(Cl)cc2[C@@](C#CC2CC2)(C(F)(F)F)O1	199.17	116
Ritonavir	CC(C)c1nc(CN(C)C(=O)NC(C(=O)NC(Cc2ccccc2)CC(O)C(Cc2ccccc2)NC(=O)OCc2cncs2)C(C)C)cs1	204.05	119
Dolutegravir	CC1CCOC2Cn3cc(C(=O)NCc4ccc(F)cc4F)c(=O)c(O)c3C(=O)N12	336.91	162
Asunaprevir	C = CC1CC1(NC(=O)C1CC(Oc2ncc(OC)c3ccc(Cl)cc23)CN1C(=O)C(NC(=O)OC(C)(C)C)C(C)(C)C)C(=O)NS(=O)(=O)C1CC1	581.77	270
Ritonavir*	CC(C)c1nc(CN(C)C(=O)N[C@H](C(=O)N[C@@H](Cc2ccccc2)C[C@H](O)[C@H](Cc2ccccc2)NC(=O)OCc2cncs2)C(C)C)cs1	609.02	283
Simeprevir*	COc1ccc2c(O[C@H]3CC4C(=O)N(C)CCCC/C = C\[C@H]5C[C@@]5(C(=O)NS(=O)(=O)C5CC5)NC(=O)[C@@H]4C3)cc(—c3nc(C(C)C)cs3)nc2c1C	826.24	356

**Table 2:** Drug-target interaction (DTI) prediction results of antiviral drugs available on markets against a novel coronavirus (SARS-CoV-2, NCBI reference sequence NC_045512.2) RNA-dependent RNA polymerase (accession YP_009725307.1). * indicates isomeric form SMILES.

Small molecules	SMILES used in the DTI prediction	*K_d_* in nM	MT-DTI Rank out of 3411
Grazoprevir*	C = C[C@H]1C[C@]1(NC(=O)[C@@H]1C[C@@H]2CN1C(=O)[C@H](C(C)(C)C)NC(=O)O[C@@H]1C[C@H]1CCCCCc1nc3ccc(OC)cc3nc1O2)C(=O)NS(=O)(=O)C1CC1	8.69	40
Ganciclovir	Nc1nc(=O)c2ncn(COC(CO)CO)c2[nH]1	11.91	58
Remdesivir*	CCC(CC)COC(=O)[C@H](C)N[P@](=O)(OC[C@@H]1[C@H]([C@H]([C@](O1)(C#N)C2 = CC = C3N2N = CN = C3N)O)O)OC4 = CC = CC = C4	20.17	93
Atazanavir	COC(=O)NC(C(=O)NC(Cc1ccccc1)C(O)CN(Cc1ccc(—c2ccccn2)cc1)NC(=O)C(NC(=O)OC)C(C)(C)C)C(C)(C)C	21.83	100
Daclatasvir	COC(=O)NC(C(=O)N1CCCC1c1ncc(—c2ccc(—c3ccc(-c4cnc(C5CCCN5C(=O)C(NC(=O)OC)C(C)C)[nH]4)cc3)cc2)[nH]1)C(C)C	23.31	107
Acyclovir	Nc1nc(=O)c2ncn(COCCO)c2[nH]1	26.66	122
Etravirine	Cc1cc(C#N)cc(C)c1Oc1nc(Nc2ccc(C#N)cc2)nc(N)c1Br	33.09	151
Entecavir*	C = C1[C@@H](n2cnc3c(=O)nc(N)[nH]c32)C[C@H](O)[C@H]1CO	52.83	215
Efavirenz*	O = C1Nc2ccc(Cl)cc2[C@@](C#CC2CC2)(C(F)(F)F)O1	76.70	287
Asunaprevir	C = CC1CC1(NC(=O)C1CC(Oc2ncc(OC)c3ccc(Cl)cc23)CN1C(=O)C(NC(=O)OC(C)(C)C)C(C)(C)C)C(=O)NS(=O)(=O)C1CC1	78.36	291
Abacavir	Nc1nc(NC2CC2)c2ncn(C3C = CC(CO)C3)c2n1	131.51	417
Darunavir*	CC(C)CN(C[C@@H](O)[C@H](Cc1ccccc1)/N = C(\O)O[C@H]1CO[C@H]2OCC[C@@H]12)S(=O)(=O)c1ccc(N)cc1	148.74	464
Dolutegravir	CC1CCOC2Cn3cc(C(=O)NCc4ccc(F)cc4F)c(=O)c(O)c3C(=O)N12	150.15	471
Rilpivirine	Cc1cc(C = CC#N)cc(C)c1Nc1ccnc(Nc2ccc(C#N)cc2)n1	228.94	628
Lomibuvir	CC1CCC(C(=O)N(c2cc(C#CC(C)(C)C)sc2C(=O)O)C2CCC(O)CC2)CC1	280.96	703
Penciclovir	Nc1nc(=O)c2ncn(CCC(CO)CO)c2[nH]1	312.93	740
Triflurdine	O = c1[nH]c(=O)n(C2CC(O)C(CO)O2)cc1C(F)(F)F	315.79	744
Nevirapine	Cc1ccnc2c1NC(=O)c1cccnc1N2C1CC1	367.50	812
Danoprevir*	CC(C)(C)OC(=O)N[C@H]1CCCCC/C = C\[C@@H]2C[C@@]2(CNS(=O)(=O)C2CC2)NC(=O)[C@@H]2C[C@@H](OC(=O)N3Cc4cccc(F)c4C3)CN2C1 = O	405.66	860
Efavirenz	O = C1Nc2ccc(Cl)cc2C(C#CC2CC2)(C(F)(F)F)O1	406.33	862
Lamivudine	Nc1ccn(C2CSC(CO)O2)c(=O)n1	511.88	981
Ritonavir	CC(C)c1nc(CN(C)C(=O)NC(C(=O)NC(Cc2ccccc2)CC(O)C(Cc2ccccc2)NC(=O)OCc2cncs2)C(C)C)cs1	624.30	1077
Saquinavir	CC(C)(C)NC(=O)[C@@H]1C[C@@H]2CCCC[C@H]2CN1C[C@@H](O)[C@H](Cc1ccccc1)NC(=O)[C@H](CC(N) = O)NC(=O)c1ccc2ccccc2n1	704.86	1146
Ganciclovir*	Nc1nc2c(ncn2COC(CO)CO)c(=O)[nH]1	723.55	1163
Raltegravir	Cc1nnc(C(=O)NC(C)(C)c2nc(C(=O)NCc3ccc(F)cc3)c(O)c(=O)n2C)o1	832.25	1241
Lamivudine*	Nc1ccn([C@@H]2CS[C@H](CO)O2)c(=O)n1	999.92	1330

**Table 3:** Drug-target interaction (DTI) prediction results of antiviral drugs available on markets against a novel coronavirus (SARS-CoV-2, NCBI reference sequence NC_045512.2) helicase (accession YP_009725308.1). * indicates isomeric form SMILES.

Small molecules	SMILES used in the DTI prediction	*K_d_* in nM	MT-DTI Rank out of 3411
Remdesivir*	CCC(CC)COC(=O)[C@H](C)N[P@](=O)(OC[C@@H]1[C@H]([C@H]([C@](O1)(C#N)C2 = CC = C3N2N = CN = C3N)O)O)OC4 = CC = CC = C4	6.48	32
Daclatasvir	COC(=O)NC(C(=O)N1CCCC1c1ncc(—c2ccc(—c3ccc(-c4cnc(C5CCCN5C(=O)C(NC(=O)OC)C(C)C)[nH]4)cc3)cc2)[nH]1)C(C)C	10.95	66
Danoprevir*	CC(C)(C)OC(=O)N[C@H]1CCCCC/C = C\[C@@H]2C[C@@]2(CNS(=O)(=O)C2CC2)NC(=O)[C@@H]2C[C@@H](OC(=O)N3Cc4cccc(F)c4C3)CN2C1 = O	19.83	113
Simeprevir*	COc1ccc2c(O[C@H]3CC4C(=O)N(C)CCCC/C = C\[C@H]5C[C@@]5(C(=O)NS(=O)(=O)C5CC5)NC(=O)[C@@H]4C3)cc(—c3nc(C(C)C)cs3)nc2c1C	23.34	122
Atazanavir	COC(=O)NC(C(=O)NC(Cc1ccccc1)C(O)CN(Cc1ccc(—c2ccccn2)cc1)NC(=O)C(NC(=O)OC)C(C)(C)C)C(C)(C)C	25.92	136
Grazoprevir*	C = C[C@H]1C[C@]1(NC(=O)[C@@H]1C[C@@H]2CN1C(=O)[C@H](C(C)(C)C)NC(=O)O[C@@H]1C[C@H]1CCCCCc1nc3ccc(OC)cc3nc1O2)C(=O)NS(=O)(=O)C1CC1	26.28	139
Asunaprevir	C = CC1CC1(NC(=O)C1CC(Oc2ncc(OC)c3ccc(Cl)cc23)CN1C(=O)C(NC(=O)OC(C)(C)C)C(C)(C)C)C(=O)NS(=O)(=O)C1CC1	28.20	151
Telaprevir	CCCC(NC(=O)C1C2CCCC2CN1C(=O)C(NC(=O)C(NC(=O)c1cnccn1)C1CCCCC1)C(C)(C)C)C(=O)C(=O)NC1CC1	40.75	194
Ritonavir	CC(C)c1nc(CN(C)C(=O)NC(C(=O)NC(Cc2ccccc2)CC(O)C(Cc2ccccc2)NC(=O)OCc2cncs2)C(C)C)cs1	41.60	196
Lopinavir*	Cc1cccc(C)c1OCC(=O)N[C@@H](Cc1ccccc1)[C@@H](O)C[C@H](Cc1ccccc1)NC(=O)[C@H](C(C)C)N1CCCNC1 = O	78.49	295
Darunavir*	CC(C)CN(C[C@@H](O)[C@H](Cc1ccccc1)/N = C(\O)O[C@H]1CO[C@H]2OCC[C@@H]12)S(=O)(=O)c1ccc(N)cc1	90.38	328
Ganciclovir	Nc1nc(=O)c2ncn(COC(CO)CO)c2[nH]1	108.21	365
Efavirenz*	O = C1Nc2ccc(Cl)cc2[C@@](C#CC2CC2)(C(F)(F)F)O1	112.69	376
Penciclovir	Nc1nc(=O)c2ncn(CCC(CO)CO)c2[nH]1	129.41	420
Etravirine	Cc1cc(C#N)cc(C)c1Oc1nc(Nc2ccc(C#N)cc2)nc(N)c1Br	175.50	522
Raltegravir	Cc1nnc(C(=O)NC(C)(C)c2nc(C(=O)NCc3ccc(F)cc3)c(O)c(=O)n2C)o1	299.81	727
Dolutegravir	CC1CCOC2Cn3cc(C(=O)NCc4ccc(F)cc4F)c(=O)c(O)c3C(=O)N12	333.32	780
Nelfinavir	Cc1c(O)cccc1C(=O)NC(CSc1ccccc1)C(O)CN1CC2CCCCC2CC1C(=O)NC(C)(C)C	365.96	823
Indinavir*	CC(C)(C)NC(=O)[C@@H]1CN(Cc2cccnc2)CCN1C[C@@H](O)C[C@@H](Cc1ccccc1)C(=O)N[C@H]1c2ccccc2C[C@H]1O	401.78	866
Efavirenz	O = C1Nc2ccc(Cl)cc2C(C#CC2CC2)(C(F)(F)F)O1	412.86	880
Entecavir*	C = C1[C@@H](n2cnc3c(=O)nc(N)[nH]c32)C[C@H](O)[C@H]1CO	452.78	943
Ritonavir*	CC(C)c1nc(CN(C)C(=O)N[C@H](C(=O)N[C@@H](Cc2ccccc2)C[C@H](O)[C@H](Cc2ccccc2)NC(=O)OCc2cncs2)C(C)C)cs1	462.20	961
Boceprevir	CC(C)(C)NC(=O)NC(C(=O)N1CC2C(C1C(=O)NC(CC1CCC1)C(=O)C(N) = O)C2(C)C)C(C)(C)C	510.35	1011
Lomibuvir	CC1CCC(C(=O)N(c2cc(C#CC(C)(C)C)sc2C(=O)O)C2CCC(O)CC2)CC1	543.41	1049
Acyclovir	Nc1nc(=O)c2ncn(COCCO)c2[nH]1	661.76	1162
Nevirapine	Cc1ccnc2c1NC(=O)c1cccnc1N2C1CC1	864.38	1373

**Table 4:** Drug-target interaction (DTI) prediction results of antiviral drugs available on markets against a novel coronavirus (SARS-CoV-2, NCBI reference sequence NC_045512.2) 3′-to-5′ exonuclease (accession YP_009725309.1). * indicates isomeric form SMILES.

Small molecules	SMILES used in the DTI prediction	*K_d_* in nM	MT-DTI Rank out of 3411
Simeprevir*	COc1ccc2c(O[C@H]3CC4C(=O)N(C)CCCC/C = C\[C@H]5C[C@@]5(C(=O)NS(=O)(=O)C5CC5)NC(=O)[C@@H]4C3)cc(—c3nc(C(C)C)cs3)nc2c1C	13.40	32
Efavirenz*	O = C1Nc2ccc(Cl)cc2[C@@](C#CC2CC2)(C(F)(F)F)O1	39.55	86
Remdesivir*	CCC(CC)COC(=O)[C@H](C)N[P@](=O)(OC[C@@H]1[C@H]([C@H]([C@](O1)(C#N)C2 = CC = C3N2N = CN = C3N)O)O)OC4 = CC = CC = C4	45.20	98
Danoprevir*	CC(C)(C)OC(=O)N[C@H]1CCCCC/C = C\[C@@H]2C[C@@]2(CNS(=O)(=O)C2CC2)NC(=O)[C@@H]2C[C@@H](OC(=O)N3Cc4cccc(F)c4C3)CN2C1 = O	49.26	108
Ganciclovir	Nc1nc(=O)c2ncn(COC(CO)CO)c2[nH]1	56.29	122
Penciclovir	Nc1nc(=O)c2ncn(CCC(CO)CO)c2[nH]1	71.76	154
Atazanavir	COC(=O)NC(C(=O)NC(Cc1ccccc1)C(O)CN(Cc1ccc(—c2ccccn2)cc1)NC(=O)C(NC(=O)OC)C(C)(C)C)C(C)(C)C	82.36	164
Entecavir*	C = C1[C@@H](n2cnc3c(=O)nc(N)[nH]c32)C[C@H](O)[C@H]1CO	82.78	165
Daclatasvir	COC(=O)NC(C(=O)N1CCCC1c1ncc(—c2ccc(—c3ccc(-c4cnc(C5CCCN5C(=O)C(NC(=O)OC)C(C)C)[nH]4)cc3)cc2)[nH]1)C(C)C	110.47	200
Grazoprevir*	C = C[C@H]1C[C@]1(NC(=O)[C@@H]1C[C@@H]2CN1C(=O)[C@H](C(C)(C)C)NC(=O)O[C@@H]1C[C@H]1CCCCCc1nc3ccc(OC)cc3nc1O2)C(=O)NS(=O)(=O)C1CC1	111.90	201
Asunaprevir	C = CC1CC1(NC(=O)C1CC(Oc2ncc(OC)c3ccc(Cl)cc23)CN1C(=O)C(NC(=O)OC(C)(C)C)C(C)(C)C)C(=O)NS(=O)(=O)C1CC1	117.26	210
Lopinavir*	Cc1cccc(C)c1OCC(=O)N[C@@H](Cc1ccccc1)[C@@H](O)C[C@H](Cc1ccccc1)NC(=O)[C@H](C(C)C)N1CCCNC1 = O	163.61	275
Ritonavir	CC(C)c1nc(CN(C)C(=O)NC(C(=O)NC(Cc2ccccc2)CC(O)C(Cc2ccccc2)NC(=O)OCc2cncs2)C(C)C)cs1	182.51	297
Lomibuvir	CC1CCC(C(=O)N(c2cc(C#CC(C)(C)C)sc2C(=O)O)C2CCC(O)CC2)CC1	182.65	298
Darunavir*	CC(C)CN(C[C@@H](O)[C@H](Cc1ccccc1)/N = C(\O)O[C@H]1CO[C@H]2OCC[C@@H]12)S(=O)(=O)c1ccc(N)cc1	195.73	313
Raltegravir	Cc1nnc(C(=O)NC(C)(C)c2nc(C(=O)NCc3ccc(F)cc3)c(O)c(=O)n2C)o1	306.99	427
Dolutegravir	CC1CCOC2Cn3cc(C(=O)NCc4ccc(F)cc4F)c(=O)c(O)c3C(=O)N12	326.89	451
Abacavir	Nc1nc(NC2CC2)c2ncn(C3C = CC(CO)C3)c2n1	346.10	466
Acyclovir	Nc1nc(=O)c2ncn(COCCO)c2[nH]1	406.63	520
Telaprevir	CCCC(NC(=O)C1C2CCCC2CN1C(=O)C(NC(=O)C(NC(=O)c1cnccn1)C1CCCCC1)C(C)(C)C)C(=O)C(=O)NC1CC1	686.16	725
Gaciclovir*	Nc1nc2c(ncn2COC(CO)CO)c(=O)[nH]1	801.41	802
Lopinavir	Cc1cccc(C)c1OCC(=O)NC(Cc1ccccc1)C(O)CC(Cc1ccccc1)NC(=O)C(C(C)C)N1CCCNC1 = O	959.76	886

**Table 5:** Drug-target interaction (DTI) prediction results of antiviral drugs available on markets against a novel coronavirus (SARS-CoV-2, NCBI reference sequence NC_045512.2) endoRNAse (accession YP_009725310.1). * indicates isomeric form SMILES.

Small molecules	SMILES used in the DTI prediction	*K_d_* in nM	MT-DTI Rank out of 3411
Efavirenz*	O = C1Nc2ccc(Cl)cc2[C@@](C#CC2CC2)(C(F)(F)F)O1	34.19	50
Atazanavir	COC(=O)NC(C(=O)NC(Cc1ccccc1)C(O)CN(Cc1ccc(—c2ccccn2)cc1)NC(=O)C(NC(=O)OC)C(C)(C)C)C(C)(C)C	50.32	77
Asunaprevir	C = CC1CC1(NC(=O)C1CC(Oc2ncc(OC)c3ccc(Cl)cc23)CN1C(=O)C(NC(=O)OC(C)(C)C)C(C)(C)C)C(=O)NS(=O)(=O)C1CC1	65.13	92
Remdesivir*	CCC(CC)COC(=O)[C@H](C)N[P@](=O)(OC[C@@H]1[C@H]([C@H]([C@](O1)(C#N)C2 = CC = C3N2N = CN = C3N)O)O)OC4 = CC = CC = C4	70.27	100
Simeprevir*	COc1ccc2c(O[C@H]3CC4C(=O)N(C)CCCC/C = C\[C@H]5C[C@@]5(C(=O)NS(=O)(=O)C5CC5)NC(=O)[C@@H]4C3)cc(—c3nc(C(C)C)cs3)nc2c1C	83.99	116
Ritonavir	CC(C)c1nc(CN(C)C(=O)NC(C(=O)NC(Cc2ccccc2)CC(O)C(Cc2ccccc2)NC(=O)OCc2cncs2)C(C)C)cs1	124.36	150
Danoprevir*	CC(C)(C)OC(=O)N[C@H]1CCCCC/C = C\[C@@H]2C[C@@]2(CNS(=O)(=O)C2CC2)NC(=O)[C@@H]2C[C@@H](OC(=O)N3Cc4cccc(F)c4C3)CN2C1 = O	235.15	247
Grazoprevir*	C = C[C@H]1C[C@]1(NC(=O)[C@@H]1C[C@@H]2CN1C(=O)[C@H](C(C)(C)C)NC(=O)O[C@@H]1C[C@H]1CCCCCc1nc3ccc(OC)cc3nc1O2)C(=O)NS(=O)(=O)C1CC1	277.87	280
Dolutegravir	CC1CCOC2Cn3cc(C(=O)NCc4ccc(F)cc4F)c(=O)c(O)c3C(=O)N12	349.63	338
Lomibuvir	CC1CCC(C(=O)N(c2cc(C#CC(C)(C)C)sc2C(=O)O)C2CCC(O)CC2)CC1	398.81	367
Lopinavir*	Cc1cccc(C)c1OCC(=O)N[C@@H](Cc1ccccc1)[C@@H](O)C[C@H](Cc1ccccc1)NC(=O)[C@H](C(C)C)N1CCCNC1 = O	472.08	415
Daclatasvir	COC(=O)NC(C(=O)N1CCCC1c1ncc(—c2ccc(—c3ccc(-c4cnc(C5CCCN5C(=O)C(NC(=O)OC)C(C)C)[nH]4)cc3)cc2)[nH]1)C(C)C	478.02	420
Efavirenz	O = C1Nc2ccc(Cl)cc2C(C#CC2CC2)(C(F)(F)F)O1	553.58	467
Darunavir*	CC(C)CN(C[C@@H](O)[C@H](Cc1ccccc1)/N = C(\O)O[C@H]1CO[C@H]2OCC[C@@H]12)S(=O)(=O)c1ccc(N)cc1	562.40	471
Nelfinavir	Cc1c(O)cccc1C(=O)NC(CSc1ccccc1)C(O)CN1CC2CCCCC2CC1C(=O)NC(C)(C)C	576.82	479
Telaprevir	CCCC(NC(=O)C1C2CCCC2CN1C(=O)C(NC(=O)C(NC(=O)c1cnccn1)C1CCCCC1)C(C)(C)C)C(=O)C(=O)NC1CC1	618.11	507
Abacavir	Nc1nc(NC2CC2)c2ncn(C3C = CC(CO)C3)c2n1	619.79	508
Raltegravir	Cc1nnc(C(=O)NC(C)(C)c2nc(C(=O)NCc3ccc(F)cc3)c(O)c(=O)n2C)o1	727.37	565
Boceprevir	CC(C)(C)NC(=O)NC(C(=O)N1CC2C(C1C(=O)NC(CC1CCC1)C(=O)C(N) = O)C2(C)C)C(C)(C)C	891.62	650

**Table 6:** Drug-target interaction (DTI) prediction results of antiviral drugs available on markets against a novel coronavirus (SARS-CoV-2, NCBI reference sequence NC_045512.2) 2′-O-ribose methyltransferase (accession YP_009725311.1). * indicates isomeric form SMILES.

Small molecules	SMILES used in the DTI prediction	*K_d_* in nM	MT-DTI Rank out of 3411
Remdesivir*	CCC(CC)COC(=O)[C@H](C)N[P@](=O)(OC[C@@H]1[C@H]([C@H]([C@](O1)(C#N)C2 = CC = C3N2N = CN = C3N)O)O)OC4 = CC = CC = C4	134.39	40
Dolutegravir	CC1CCOC2Cn3cc(C(=O)NCc4ccc(F)cc4F)c(=O)c(O)c3C(=O)N12	153.73	46
Atazanavir	COC(=O)NC(C(=O)NC(Cc1ccccc1)C(O)CN(Cc1ccc(—c2ccccn2)cc1)NC(=O)C(NC(=O)OC)C(C)(C)C)C(C)(C)C	390.67	90
Efavirenz*	O = C1Nc2ccc(Cl)cc2[C@@](C#CC2CC2)(C(F)(F)F)O1	423.00	99
Boceprevir	CC(C)(C)NC(=O)NC(C(=O)N1CC2C(C1C(=O)NC(CC1CCC1)C(=O)C(N) = O)C2(C)C)C(C)(C)C	433.93	101

Among the prediction results, atazanavir was predicted to have a potential binding affinity to bind to RNA-dependent RNA polymerase (*K_d_* 21.83 nM), helicase (*K_d_* 25.92 nM), 3′-to-5′ exonuclease (*K_d_* 82.36 nM), 2′-O-ribose methyltransferase (*K_d_* of 390.67 nM), and endoRNAse (*K_d_* 50.32 nM), which suggests that all subunits of the COVID-19 replication complex may be inhibited simultaneously by atazanavir (Table 2, Table 3, Table 4, Table 5, Table 6). Also, ganciclovir was predicted to bind to three subunits of the replication complex of the COVID-19: RNA-dependent RNA polymerase (*K_d_* 11.91 nM), 3′-to-5′ exonuclease (*K_d_* 56.29 nM), and RNA helicase (*K_d_* 108.21 nM). Lopinavir and ritonavir, active materials of AbbVie’s Kaletra, both were predicted to have a potential affinity to COVID-19 helicase (Table 3) and are suggested as potential MERS therapeutics [28]. Recently, approximately $2 million worth of Kaletra doses were donated to China [29], and a previous clinical study of SARS by Chu et al. [30] may support this decision [30]. Another anti-HIV drug, Prezcobix of Johnson & Johnson, which consists of darunavir and cobicistat, was to be sent to China [29], and darunavir is also predicted to have a *K_d_* of 90.38 nM against COVID-19′s helicase (Table 3). However, there was no current supporting literature found for darunavir to be used as a CoV therapeutic. Although remdesivir is not a FDA approved drug, its predicted potency to COVID-19 resulted as follows: against RNA-dependent RNA polymerase (*K_d_* 20.17 nM), helicase (*K_d_* 6.48 nM), 3′-to-5′ exonuclease (*K_d_* 45.20 nM), 2′-O-ribose methyltransferase (*K_d_* of 134.39 nM), and endoRNAse (*K_d_* 70.27 nM).

## 4 Discussion

In many cases, DTI prediction models serve as a tool to repurpose drugs to develop novel usages of existing drugs. The application of DTI prediction in the present study may be useful to control unexpected and rapidly spreading infections such SARS-CoV, Middle East respiratory syndrome (MERS-CoV), and SARS-CoV-2 at the frontline of the disease control until better therapeutic measures are developed.

Several recent studies have identified promising drug candidates that may help reduce symptoms of COVID-19 by inhibiting some aspects of SARS-CoV-2. For example, remdesivir and chloroquine showed inhibitory effects against SARS-CoV-2 *in vitro* [31]. Another *in-vitro* study showed that hydroxychloroquine was found to be more potent than chloroquine for inhibiting SARS-CoV-2 [32]. Remdesivir and lopinavir/ritonavir (Kaletra) also reduced pneumonia-associated symptoms of some COVID-19 patients [33], [34]. However, these studies are based on previous knowledge that these drugs showed some inhibitory effects on similar coronaviruses such as SARS-CoV and/or MERS-CoV. In contrast, our approach was truly based on a pre-trained MT-DTI deep-learning model that understands drug-target interactions without domain knowledge [12]. In fact, MT-DTI successfully identified the epidermal growth factor receptor (EGFR)-targeted drugs that are used in clinics (in top-30 predicted candidates) among 1794 chemical compounds registered in the DrugBank database in a previous study [12], suggesting that 3D structural information of proteins and/or molecules is not necessarily required to predict drug-target interactions.

Our results showed the following intriguing findings that need to be tested experimentally and clinically in the near future. First, MT-DTI generally showed similar results overall compared to the conventional 3D structure-based prediction model, AutoDock Vina, but some differences were observed. For example, atazanavir, remdesivir, and efavirenz were the top three predicted drugs that may bind to the 3C-like proteinase of SARS-CoV-2. This is while saquinavir, nelfinavir, and grazoprevir were the top three drugs identified by AutoDock Vina (Fig. 1). Secondly, when the search space was expanded to all FDA-approved drugs, some immunosuppressant drugs (rapamycin and everolimus) and a drug (tiotropium bromide) for asthma and chronic obstructive pulmonary disease (COPD) were identified as promising candidates by MT-DTI. In contrast, AutoDock Vina predicted purmorphanime, lumacaftor, and verrucarin A were the top three drugs that could bind to the 3C-like proteinase of SARS-CoV-2. However, there is currently no supporting evidence that these drugs may be effective in inhibiting SARS-CoV-2. Lastly, atazanavir appears to be effective in the treatment of COVID-19 by showing overall high binding affinities among tested antivirals for six proteins of SARS-CoV-2 including 3C-like proteinase and the replication complex components (Table 1, Table 2, Table 3, Table 4, Table 5, Table 6, Table 6 and S1–6). But, this prediction also needs to be validated *in vitro*, *in vivo*, and in a wide range of clinical trials for efficacy and safety.

We hope our prediction results may support experimental therapeutic options for China and other countries suffering from the SARS-CoV-2 pandemic and align with recent clinical trials [35].

## CRediT authorship contribution statement

**Bo Ram Beck:** Data curation, Writing - original draft. **Bonggun Shin:** Conceptualization, Methodology, Software. **Yoonjung Choi:** Methodology, Writing - review & editing. **Sungsoo Park:** Conceptualization, Methodology, Data curation, Software. **Keunsoo Kang:** Conceptualization, Methodology, Writing - original draft, Writing - review & editing.

## Declaration of Competing Interest

Beck B.R., Choi Y., and Park S. are employed by company Deargen Inc. Shin B. is employed by Deargen Inc as a part-time advisor. Kang K. is one of the co-founders of, and a shareholder in, Deargen Inc.
